# The Establishment of a Primary Culture System of Proximal Tubule Segments Using Specific Markers from Normal Mouse Kidneys

**DOI:** 10.3390/ijms13045098

**Published:** 2012-04-23

**Authors:** Masumi Kamiyama, Michelle K. Garner, Kristina M. Farragut, Hiroyuki Kobori

**Affiliations:** Department of Physiology, Hypertension and Renal Center of Excellence, Tulane University Health Sciences Center, 1430 Tulane Avenue, LA 70112, USA; E-Mails: mkamiyam@tulane.edu (M.K.); mgarner@tulane.edu (M.K.G.); kfarragu@tulane.edu (K.M.F.)

**Keywords:** primary culture, proximal convoluted tubules, proximal straight tubules, angiotensinogen, intrarenal renin-angiotensin system

## Abstract

The proximal tubule contains the highest expression of angiotensinogen mRNA and protein within the kidney and plays a vital role in the renal renin-angiotensin system. To study the regulation of angiotensinogen expression in the kidney in more detail, the proximal tubule needs to be accurately isolated from the rest of the nephron and separated into its three segments. The purpose of this study was to design a novel protocol using specific markers for the separation of proximal tubule cells into the three proximal tubule segments and to determine angiotensinogen expression in each segment. Kidneys were removed from C57BL/6J mice. The proximal tubules were aspirated from region of a Percoll gradient solution of the appropriate density. The proximal tubule was then separated into its three segments using segment-specific membrane proteins, after which each segment was characterized by a different specific marker (sodium-glucose transporter 2 for Segment 1; carbonic anhydrase IV for Segment 2; ecto-adenosine triphosphatase for Segment 3). The isolation of proximal tubules into three segments was successful, and angiotensinogen mRNA in Segment 2 and 3 and angiotensinogen protein in all three segments were confirmed. This protocol will be helpful for future studies of the detailed mechanisms of the intrarenal renin-angiotensin system.

## 1. Introduction

Several methods for the primary culture of proximal tubules have been described [1–13]. The macroseparation technique was used to establish a primary culture of proximal tubules using a Percoll density gradient and serum-free media [1–4]. Hormonally defined serum-free media has been used to prepare primary cultures of differentiated cells that lack fibroblast overgrowth, including primary kidney tubule epithelial cells [5–8]. The microdissection technique has been used for the separation of proximal tubules for primary culture, as well as for the study of specific segments [9–11]. Additionally, the isolation of proximal tubules from urine has been reported [12,13]. However, these methods of primary culture of proximal tubules and of proximal tubule segments have been associated with problems in terms of the specificity of cells, cell condition, and proliferation in culture.

The importance of the renin-angiotensin system (RAS) in the regulation of blood pressure and fluid and electrolyte homeostasis is well recognized [14]. Recently, the focus of interest on the RAS has shifted towards understanding the local RAS in specific tissues [14–16]. In particular, the role of renal RAS is distinct because all of the necessary components are localized in the nephron of the kidney [14]. We previously observed that angiotensinogen (AGT) mRNA was expressed largely in the proximal tubules and also demonstrated that AGT protein in the kidney is predominantly localized in the proximal tubules [14,17,18]. Moreover, others and we showed that intrarenal AGT mRNA and/or protein levels are increased in various kidney diseases including diabetic nephropathy, immunoglobin A nephropathy, and radiation nephropathy [18,19].

Structurally, the proximal tubules can be divided into three segments; comprising the pars convoluta, Segment (S)1 segment, the end of the pars convoluta, S2 segment, and the pars recta, S3 segment [20]. Although each segment has different functions, the level and localization of the expression of AGT mRNA and protein in each segment, and the role that changes in the expression of AGT may play in the development of kidney diseases remain unclear. Therefore, the technique of primary culture of proximal tubule three segments and new data of detailed localization of AGT mRNA and protein in the proximal tubule segments is required.

The purpose of this study was to design a protocol to culture pure proximal tubule cells using a separation step that employs magnetic particles. Furthermore, we established a protocol for the separation of proximal tubule cells into those from segments S1, S2, and S3, and for analysis of AGT mRNA and protein in each of these three types of cell.

## 2. Results and Discussion

### 2.1. Primary Culture of Proximal Tubules

**Figure 1:**
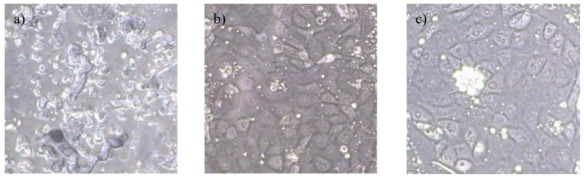
(**a**) Isolated proximal tubules aspirated from a region of the appropriate density (Day 0); (**b**) Proximal tubule cells on day 4 of culture; (**c**) Purified proximal tubule cells (200×, Day 4).

On the day of isolation of the proximal tubules (day 0), many fragments of tubule shape were seen in the samples aspirated from the appropriate region of the density gradient containing the centrifuged cortical solution [2], as seen in Figure 1a. On day 4 of the cell culture (Figure 1b) successful cultures were identified by the attachment of cells to the culture dish as well as the proliferation of multiple cell colonies. After using the EasySep^®^ kit and biotin-conjugated anti-Tamm-Horsfall glycoprotein (THG) antibody (a marker of early distal convoluted tubules [21]) to eliminate contamination with distal tubules, the cultured proximal tubule cells were purified on day 4, as shown in Figure 1c.

### 2.2. Characterization of Proximal Tubules

**Figure 2:**
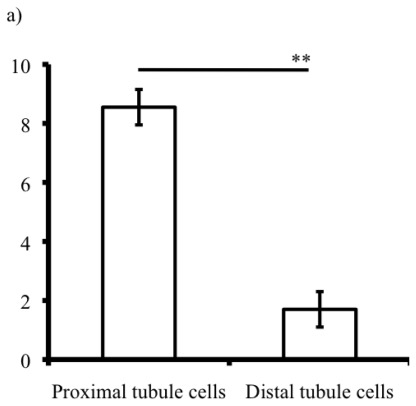

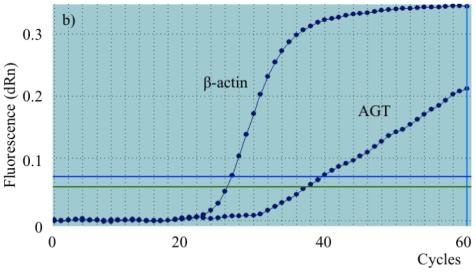
(**a**) Alkaline phosphatase activity of tubule cells. ** *P* < 0.01 *vs*. distal tubule cells; (**b**) AGT (angiotensinogen) expression in the proximal tubule cells. Values were normalized to β-actin mRNA expression with a final result of 0.34 (AGT/β-actin ratio).

To characterize the purified proximal tubules, we examined alkaline phosphatase activity (the proximal tubule brush border enzyme) [3,11] and AGT mRNA expression (we reported previously that AGT was highly expressed in the proximal tubules) [17]. Alkaline phosphatase activity was higher in the proximal tubules (8.6 ± 0.6 ng/μg) than in the separated samples from the 2^nd^ density band, presumed to contain the distal tubules (1.7 ± 0.3 ng/μg) (Figure 2a). Quantitative real-time RT-PCR shows that AGT mRNA was expressed in the proximal tubules (Figure 2b). Samples from the 2^nd^ density band, presumed to be distal tubules, did not express AGT mRNA (data not shown).

**Figure 3:**
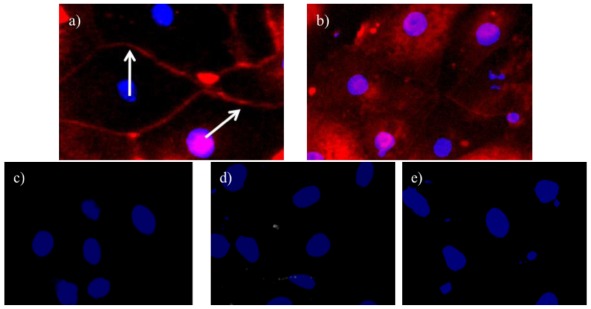
Proximal tubule cells stained for (**a**) CD13 (aminopeptidase N); (**b**) AGT (Angiotensinogen); (**c**) AQP-2 (aquaporin-2); (**d**) AE-1 (anion exchanger type-1), and (**e**) Thy1.1 (thymus cell antigen-1, theta, 600×). Red, markers; Blue, nuclei. Arrows indicate cell membrane.

Because we could confirm the isolation of proximal tubules from a nephron, we further characterized the proximal tubule cells using positive and negative markers. Immunocytochemical analysis of the proximal tubule cells showed staining for aminopeptidase N (CD13, a marker of proximal tubules) [22], exhibited by the red color in the cell membranes in Figure 3a. Staining was also positive for AGT protein expression in the cytosol of the cells [17], shown by the red color in Figure 3b.

On the other hand, the cells did not express aquaporin-2 (AQP-2; a marker of the distal tubules and the principle collecting duct) [23,24], anion exchanger type-1 (AE-1; a marker of the intercalated correcting duct) [23], or thymus cell antigen-1, theta (Thy1.1; a marker of mesangial cells) [25], as exhibited by the absence of red color in Figures 3c,d, and e, respectively.

### 2.3. Separation and Characterization of Proximal Tubule Cells from the S1, S2, and S3 Segments

The EasySep method, which makes use of antibodies specific for glucose transporter 5 (GLUT5) to identify S3 cells and solute carrier family 36, member 1 (SLC36A1) to identify S1 cells, was then used to isolate these cell types. The segmental separation in this study was successful as demonstrated by the positive staining for sodium glucose transporter 2 (SGLT2) in S1 cells [20], carbonic anhydrase IV (CA IV) in S2 cells [20], and ecto-adenosine triphosphatase (ecto-ATPase) in S3 cells [20].

**Figure 4:**
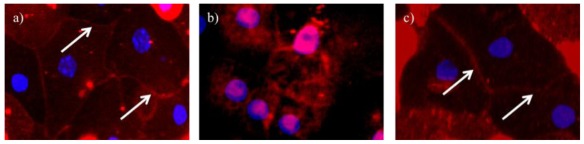
(**a**) S (Segment)1 cells stained for SGLT-2 (sodium glucose transporter 2, 600×); (**b**) S2 cells stained for CA IV (carbonic anhydrase IV, 600×); (**c**) S3 cells stained for ecto-ATPase (ecto-adenosine triphosphatase, 600×). Red, markers; Blue, nuclei. Arrows indicate cell membrane.

The isolated S1 cells stained positive for SGLT2, as observed by the red color of the cell membrane (Figure 4a). The isolated S2 cells in Figure 4b stained positive for CAIV, as indicated by the bright red color in the cytosol. The isolated S3 cells stained positive for ecto-ATPase, as shown in Figure 4c by the red color in the cytosol of the cells.

### 2.4. AGT mRNA and Protein Expression in S1, S2, and S3 Proximal Tubule Cells

**Figure 5:**
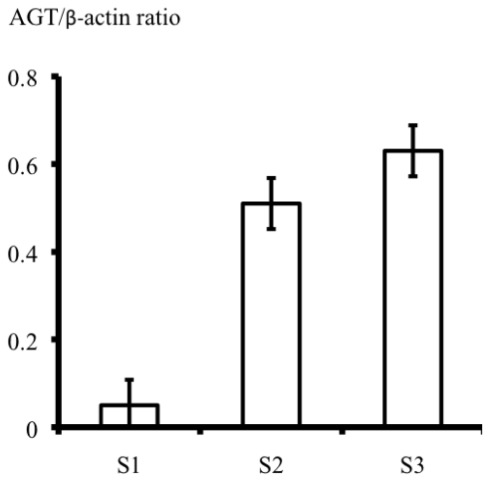
Quantitative real-time RT-PCR results of AGT (angiotensinogen) mRNA expression in S (Segment)1, S2, and S3 cells.

Quantitative real-time RT-PCR performed on cells from each proximal tubule segment (Figure 5), revealed that the S1 cells expressed little AGT mRNA, whereas both S2 and S3 cells expressed much greater levels of AGT mRNA.

**Figure 6:**
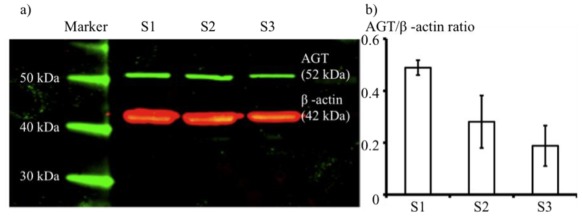
Western blot analysis of AGT (angiotensinogen) and β-actin protein expression (**a**) and quantitated AGT protein expression ratio (**b**).

Western blot analysis of cells from the three segments revealed expression of AGT protein in each of S1, S2, and S3, as shown in Figure 6a by the solid green bands, which correspond to a size of 52 kDa, the correct mass for the AGT protein. AGT expression values were normalized to those of β-actin (red bands, which are at 42 kDa) providing relative levels of 0.48 ± 0.03 for S1, 0.28 ± 0.10 for S2, and 0.19 ± 0.08 for S3 (Figure 6b).

### 2.5. Discussion

A few reports have described primary culture systems for proximal tubules [1–13]. However, such cultures can be contaminated with other cell types. Furthermore, the low proliferative capacity of the proximal tubule cells has added to the difficulty of establishing successful cultures. To purify specific proximal tubule cells quickly and accurately, we utilized a separation procedure that makes use of the EasySep^®^ step system employing specific cell markers [26,27].

Using this method, we isolated mouse kidney proximal tubules that tested positive for CD13 [22] and AGT mRNA and protein [17], and negative for AQP2 [23,24], AE-1 [23], and Thy-1.1 [25]. These results indicate that the cultured cells were successfully isolated as proximal tubule cells and these cells were not contaminated another cells, distal tubules, collecting duct, and mesangial cells. Also, all cultures were examined under phase-contrast light microscopy throughout the culture period, and fibroblasts were not detected (data not shown), thus giving further credibility to the Percoll density gradient macroseparation method previously reported.

Similarly, the segmental separation of proximal tubule cells was successful as demonstrated by the positive staining for SGLT2 in S1 cells [20], CAIV in S2 cells [20], and ecto-ATPase in S3 cells [20]. The EasySep^®^ method, which used antibodies against plasma membrane proteins (GLUT5 to identify S3 cells [28] and SLC36A1 to identify S1 cells [29]), is a useful tool for the separation of proximal tubule cells.

Recent reports have described more details on the localization of RAS components in kidneys [30]. AGT mRNA is expressed largely in the proximal tubules, and only small amounts are expressed in glomeruli and vasa recta. Intrarenal AGT is formed primarily in the proximal tubule cells and then it is secreted into the tubular fluid [31]. There are some reports describing AGT localization in proximal tubules; however, it is still controversial as to which proximal tubule segments express AGT [32–36]. Once the proximal tubule segments were successfully separated, the study inquired further into the expression of AGT mRNA and protein within each segment, S1, S2, and S3. These results revealed that AGT mRNA was expressed in S2 and S3 but not in S1. On the contrary, AGT protein expression was strong in S1 and also in S2 and S3. An our recent study produced noticeably similar results [37]. In the study, a multiple-staining method using antibodies including SGLT2, CAIV, and ecto-ATPase was used on kidney tissue from normal rats in order to delineate the localization of AGT mRNA and protein in renal proximal tubules. The staining determined that AGT mRNA was expressed primarily in the S3 segments with weak AGT mRNA expression in the S2 segments. In contrast, AGT protein was strongly expressed in the S1 segments, and was also present in the S2 and S3 segments.

Data from the current study and subsequent study clearly show that the main localization of AGT mRNA and AGT protein are different. These results suggest that AGT mRNA transcription and AGT protein synthesis and metabolism are performed in different proximal tubule segments. The metabolic processes of AGT in the proximal tubule are extremely complex, and the details have not been fully discovered. However, the present data provide some insight in showing that AGT mRNA is transcribed primarily in S2 and S3. In terms of AGT protein, expression was mainly seen in S1. This finding is congruent with the previous report that AGT is a ligand of megalin, which is expressed in S1 [34,38]. A possible explanation to support this result is that the binding of AGT to specific membrane receptors like megalin as well as endocytosis of these components may take place in S1. The uncertainty regarding the supplying source of AGT protein in S1 is not resolved from our results. It has been reported that AGT mRNA and protein are expressed not only in proximal tubules but also weakly in the glomeruli, specifically in mesangial cells [25]. From the analysis of this data and other reports, it is possible that AGT protein is generated in mesangial cells, and transported to S1 of the proximal tubule by endocytosis. However, further discussion and research is still needed on the detailed mechanism of translation, secretion, metabolism, and degradation of AGT protein in the kidney.

Moreover, we have shown that AGT expression in these segments was increased in diabetic rats [39]. Thus, AGT is an important factor of the proximal tubules, as well as displaying distinctive quantitative features in several diseases. In a future research, the present protocol will be helpful for studies of the detailed mechanisms of the intrarenal renin-angiotensin system.

## 3. Experimental Section

### 3.1. Isolation of Kidney Tubules

All procedures and protocols used in this study were approved by the Institutional Animal Care and Use Committee of the Tulane University Health Sciences Center. Male 18-week-old C57BL/6J mice obtained from Dr. Sigmund, University of Iowa were used for this study [40]. All mice were sacrificed using a guillotine, and kidneys were immediately harvested and washed in sterile ice-cold and 95% O_2_/5% CO_2_ equilibrated Krebs-Henseleit saline (KHS, pH 7.4) containing the following: 119 mmol/L NaCl, 4.7 mmol/L KCl, 1.9 mmol/L CaCl_2_, 1.2 mmol/L KH_2_PO_4_, 1.2 mmol/L MgSO_4_·7H_2_O, and 25 mmol/L NaHCO_3_. Each kidney was decapsulated, bisected, and the inner medullary portion was excised. The remaining cortical and outer medullary regions were pulverized, and mixed in a solution of 30 mL of KHS containing 1 mg/mL collagenase (type I; Invitrogen, Carlsbad, CA, USA) for every 1 g of minced kidney. The solution was bubbled with 95% O_2_/5% CO_2_ during incubation for 30 min at 37 °C. The solution was filtered through a 210 μm mesh sieve (Fisher Scientific, Houston, TX, USA). To centrifuge tubes (Sorvall Products, Newtown, CT, USA) containing 30 mL of 45% Percoll (Sigma Chemical Co, St Louis, MO, USA) solution and 5 mL of 90% Percoll solution, 5 mL of the kidney solution was slowly added to the top of the tube [1–3]. The tubes were centrifuged in a Sorvall centrifuge (Sorvall Products, Newtown, CT, USA) using a rotor (Sorvall model: ss-34) at 20,000 g and 4 °C for 30 min as described previously [1–3]. Centrifugation resulted in the separation of 4 banded layers. The solution within the 3^rd^ layer containing the proximal tubules, was aspirated and centrifuged at 1500 rpm at room temperature for 2 min to remove the Percoll solution.

### 3.2. Primary Culture of Proximal Tubule Cells

The cells were cultured in serum-free hormonally defined media sequentially as described by Chung *et al*. [8]. The basal culture medium used is as follows; Dulbecco’s modified Eagle’s (DME) medium (Invitrogen, Carlsbad, CA, USA), supplemented with 2 mmol/L glutamine, 100 IU/mL penicillin, 100 μg/mL streptomycin, 5 μg/mL insulin, 5 × 10^8^ mol/L hydrocortisone, 5 μg/mL transferring, 2 mmol/L butyrate, 2 mmol/L alanine, and 2 mmol/L lactate. The bicarbonate concentration was adjusted to 24 mmol/L to maintain the medium at pH 7.4 to 7.5. The proximal tubules were placed in wells with 5 mg of cell protein for every 5 mL of culture medium. After 48 h of the culture, the medium was changed, and every two days thereafter.

### 3.3. Separation of Proximal Tubule Cells

After the primary culture of proximal tubules were sub-confluent (day 4), the cells were harvested with trypsin-EDTA (Invitrogen, Carlsbad, CA, USA), and contaminating distal tubule cells were eliminated with biotin-conjugated anti-THG antibody (marker of early distal convoluted tubules; Santa Cruz Biotechnology Inc., Santa Cruz, CA, USA), EasySep magnetic particles^®^ (StemCell Technologies Inc., Vancouver, BC, Canada), and EasySep magnet^®^ [27]. Biotin labeling of anti-THG antibody was performed using Biotin Labeling Kit-NH_2_^®^ according to the manufacturer’s protocol (Abnova, Walnut, CA, USA).

Purified proximal tubule cells were further separated into three kinds of cells, *i.e*., proximal tubule cells from S1, S2, or S3 segments. Biotin labeling of anti-GLUT5 antibody and anti-SLC36A1 was performed using a Biotin Labeling Kit-NH_2_. Proximal tubule cells from S3 segments were separated by EasySep^®^ kit using biotin-conjugated anti-GLUT5 antibody, a S3-specific marker [28]. The remaining cells included proximal tubule cells from S1 and S2 segments. We further separated these remaining cells by the EasySep^®^ kit using biotin-conjugated anti-SLC36A1 antibody, a S1-specific marker [29]. Proximal tubule cells from S1 segments were obtained, and the remaining cells were considered to be proximal tubule cells from S2 segments. Each of the proximal tubule cell fractions (from S1, S2 and S3 segments) was cultured in the above-mentioned culture medium.

### 3.4. Alkaline Phosphatase Activity

Using proximal tubules, we investigated alkaline phosphatase activity (the proximal tubule brush border enzyme) [3,11] using SensoLyte^®^ *p*NPP Alkaline Phosphatase Assay Kit according to the manufacturer’s instructions (AnaSpec, Fremont CA, USA). Alkaline phosphatase activity was normalized to the protein concentration of cellular lysates. A Micro BCA Protein Assay kit was used for the protein assay (Pierce Chemical Co, Rockford, IL, USA) [25,41].

### 3.5. Immunocytochemistry

We used immunocytochemistry to evaluate the expression of CD13, a marker of proximal tubules [22], AGT, a marker of proximal tubules [17], AQP-2; a marker of distal tubules and the principle collecting duct [23,24], AE-1; a marker of the intercalated correcting duct [23], and Thy1.1; a marker of mesangial cells [25] in the proximal tubule cells. For the characterization of proximal tubule cells from S1, S2, and S3 segments, we also performed immunocytochemistry with the segment-specific markers, SGLT2 for S1 [20], CA IV for S2 [20], and ecto-ATPase for S3 [20].

We used rabbit polyclonal anti-CD13 antibody (1:100; Santa Cruz Biotechnology, Santa Cruz, CA, USA), rabbit polyclonal anti-AGT antibody (1:100; gift from Immuno-Biological Laboratories Co. Ltd., Gunma, Japan), rabbit polyclonal anti-AQP2 antibody (1:100; gift from Dr. Alexis A. Gonzalez, Tulane University Health Sciences Center), rabbit polyclonal anti-AE-1 antibody (1:100; gift from Dr. Alexis A. Gonzalez, Tulane University Health Sciences Center), rabbit monoclonal anti-Thy-1 antibody (1:100; Serotec, Kidlington, UK), rabbit polyclonal anti-SGLT2 antibody (1:100; Santa Cruz Biotechnology, Santa Cruz, CA, USA), goat polyclonal anti-CA IV antibody (1:100; Santa Cruz Biotechnology, Santa Cruz, CA, USA), and rabbit polyclonal anti-ecto-ATPase antibody (1:100; Santa Cruz Biotechnology, Santa Cruz, CA, USA).

After washing cells, 2 μg/mL Alexa Fluor^®^ 488 (goat anti-rabbit IgG or donkey anti-goat IgG; Invitrogen, Carlsbad, CA, USA) was used as secondary antibody and we also stained nuclei with DAPI (4, 6-diamino-2- phenylindole; Invitrogen, Carlsbad, CA, USA).

The staining was observed with fluorescence microscopy (BX51; Olympus, Tokyo, Japan).

### 3.6. Quantitative Real-Time RT-PCR

RNAs were extracted from the tissues using the RNeasy Mini kit according to the manufacturer’s instructions (QIAGEN, Valencia, CA, USA). Expression of the AGT gene in the proximal tubules and proximal tubule three segments (proximal tubules were sub-cultured twice to purify proximal tubule three segments) was examined using the Brilliant II QRT-PCR Master Mix kit, 1-Step (Stratagene, La Jolla, CA, USA) following the RT-PCR conditions according to manufacturer’s instructions. Quantitative real-time RT-PCR was performed as previously described [42,43]. Data from quantitative real-time RT-PCR were normalized to β-actin mRNA expression. Primer sequences were as follows: AGT, forward primer 5′-TAT CCA CTG ACC CAG TTC TT-3′, reverse primer 5′-AAG TGA ACG TAG GTG TTG AAA-3′, probe 5′-/6-FAM/CTG TGA CAG GGT GGA AGA TGA ACT TGC CA/3BHQ-1/3′; β-actin, forward primer 5′-ATC ATG AAG TGT GAC GTT GA-3′, reverse primer 5′-GAT CTT CAT GGT GCT AGG AGC-3′, probe 5′-/6-FAM/TCT ATG CCA ACA CAG TGC TGT CTG GT/3BHQ-2/3′.

### 3.7. Western Blot

Using proximal tubule three segments (proximal tubules were sub-cultured twice to purify proximal tubule three segments), western blot analysis was performed as previously described [22] using the Odyssey^®^ Infrared Imaging System (LI-COR Biosciences, Lincoln, NE, USA) for identification of AGT [25,41]. Rabbit polyclonal anti-AGT antibody, mouse anti-β-actin antibody (LI-COR Biosciences, Lincoln, NE, USA), and appropriate secondary antibodies (LI-COR Biosciences) were used. Quantification of images of AGT protein levels was normalized to β-actin protein levels.

### 3.8. Statistical Analysis

All data are presented as the mean ± SEM. Alkaline phosphatase activities were evaluated by the *t*-test. *P* < 0.05 was considered to be statistically significant. All of the computations, including data management and statistical analyses, were performed with JMP software (SAS Institute, Cary, NC, USA).

## 4. Conclusions

The activation of the intrarenal RAS has been recognized as a possible mechanism of kidney diseases including diabetic nephropathy, immunoglobin A nephropathy, and radiation nephropathy. Within the RAS, AGT is an important biomarker in the activation of RAS. General localization of AGT has shown its dominant presence in the proximal tubule of the nephron. This study establishes a novel method to isolate the three segments of the proximal tubule and to more precisely assess the location of AGT mRNA and protein expression. In the future, these methods may be useful to investigate the segmental regulation of AGT mRNA and protein expression in physiological and pathological conditions such as diabetic nephropathy.
